# Impact of the final adjective in the Medical Student Performance Evaluation on determination of applicant desirability

**DOI:** 10.1080/10872981.2018.1542922

**Published:** 2018-11-08

**Authors:** Mark A. Ward, Debra L. Palazzi, Martin I. Lorin, Anoop Agrawal, Hilel Frankenthal, Teri L. Turner

**Affiliations:** a Department of Pediatrics, Baylor College of Medicine, Houston, TX, USA; b Department of Medicine, Baylor College of Medicine, Houston, TX, USA; c Department of Child Health, University of Missouri Hospital and Clinics, St. Louis, MO, USA

**Keywords:** Medical Student Performance Evaluation, dean’s letter, medical student, intern selection, residency applicants

## Abstract

**Background**: The Medical Student Performance Evaluation (MSPE) is a primary source of information used by residency programs in their selection of trainees. The MSPE contains a narrative description of the applicant’s performance during medical school. In 2002, the Association of American Medical Colleges’ guideline for preparation of the MSPE recommended inclusion of a comparative summative assessment of the student’s overall performance relative to his/her peers (*final adjective*).

**Objective**: We hypothesize that the inclusion of a final adjective in the MSPE affects a reviewer’s assessment of the applicant’s desirability more than the narrative description of performance and designed a study to evaluate this hypothesis.

**Design**: Fifty-six faculty members from the Departments of Pediatrics and Medicine with experience reviewing MSPEs as part of the intern selection process reviewed two pairs of mock MSPE letters. In each pair, the narrative in one letter was superior to that in the other. Two final adjectives describing relative class ranks were created. Each subject was first presented with a pair of letters with mismatched final adjective (study), *i.e*., the letter with the stronger narrative was presented with the weaker final adjective and vice versa. The subject was then presented with a second pair of letters without final adjectives (control). Subjects ranked the relative desirability of the two applicants in each pair.

**Results**: The proportion of rankings congruent with the strength of the narratives under study and control conditions were compared. Subjects were significantly less likely to rank the applicants congruent with the strength of the narratives when the strength of the final adjectives conflicted with the strength of the narrative; 42.9% of study letters were ranked congruent with the narrative versus 82.1% of controls (*p* = 0.0001).

**Conclusion**: The MSPE final adjective had a greater impact than the narrative description of performance on the determination of applicant desirability.

**Abbreviations**: MSPE: Medical Student Performance Evaluation; AAMC: Association of American Medical Colleges; BCM: Baylor College of Medicine

## Introduction

Residency programs consider multiple factors when ranking applicants for the National Residency Match Program. Program directors have indicated that the Medical Student Performance Evaluation (MSPE) is an important factor used by 94% of directors [1]. The MSPE, formerly known as the ‘dean’s letter,’ aims to provide a comprehensive assessment for residency program directors of a student’s noteworthy characteristics, professional behaviors, salient experiences, and academic achievements.

In 2002, in response to concerns about the value of the dean’s letter, the Association of American Medical Colleges (AAMC) published guidelines for the letter, including changing the name to the MSPE to reflect its purpose as an evaluation of the student’s performance rather than a recommendation [2]. These guidelines recommended a specific format, which included the traditional narratives describing performance during clinical rotations and a summative assessment of the student’s overall performance relative to his or her peers if a school-wide comparison of the applicant is made. The 2016 recommendations of the AAMC refer to this assessment as the *final adjective* or the *overall rating* [3]. This term has often informally been referred to as the bottom line. Examples of final adjectives include ‘outstanding,’ ‘superior,’ ‘very good,’ and ‘qualified.’ Currently there is no standard set of comparative descriptors used by every US medical school. The 2016 AAMC statement also recommends that if a final adjective is used, the MSPE should list the full range of descriptors and the percentage of students falling into each comparison group [3]. However, if a medical school does not provide a school-wide comparison then the final adjective or overall rating should not be included.

Mallott suggested that many program directors focus primarily or solely on the final adjective when evaluating the MSPE [4]. While studies have looked at the value placed on clinical grades contained in the MSPE compared to class rank [5,6], there are no studies assessing the weight given to the *narrative description* of clinical performance versus the *final adjective* by faculty members when ranking applicants. Currently, within the same applicant pool for any given year, some MSPEs contain final adjectives and others do not. The purpose of our study was to determine the impact of the narrative description of clinical performance compared to the final adjective on the reviewer’s assessment of applicant desirability.

## Methods

We designed a post-test only study in which subjects served as their own controls. Subjects were full-time faculty members of the Department of Pediatrics or the Department of Medicine at Baylor College of Medicine (BCM). All subjects were serving or had recently served on their respective departments’ resident selection committees and had experience reviewing MSPEs. Participation was voluntary. After ranking the letters, participants completed a demographic survey that included a question as to whether or not they felt rushed during the study. This question was included as a surrogate measure to assess whether or not the faculty had sufficient time to read and reflect critically on the MSPEs. The study was conducted over a 6-month period.

One of the authors (MIL), with over 30 years of experience reviewing dean’s letters and MSPEs, created two pairs (pair AB and pair CD) of mock narrative descriptions of clinical performance (without grades), such that one narrative in each pair was superior to that of the other: narrative A was superior to B and narrative C was superior to D. Then, without being told which were the stronger applicants, the other five authors read and ranked A against B and C against D. Revisions of the narratives were made (by MIL) until the other authors reached consensus as to correct rank order within each pair, consensus meaning that at least four of the five authors correctly identified the stronger applicant (see Appendix).

The authors then created two final adjectives describing the applicant as ‘outstanding’ or ‘excellent’ followed by an explanation that ‘outstanding’ indicated that the applicant ranked in the top 20% and ‘excellent’ indicated that he or she ranked in the second 20% of students at that school. Each letter also included the full range of descriptors and the percentage of students falling into each comparison group. The outcome measure assessed was whether or not the faculty member correctly (congruent with strength of narratives) assessed the ranking of each pair of MSPEs both with and without a final adjective. To minimize implicit bias, the gender of the students was the same in all letters and the name of the medical school was not stated. Student names were ethnically similar. None of the students deviated from the expected graduation date, remediated coursework, or were the recipient of any adverse action(s). Each MSPE contained a short description of unique characteristics (2016 AAMC recommendations now called ‘noteworthy’ characteristics) and similar preclinical/basic science curricular performance and United States Medical Licensing Examination Step 1 scores.

Faculty members were instructed that they would be presented with two pairs of MSPEs representing four unique students from four different medical schools. They were to review the first pair of letters and determine which of the two students was the more desirable applicant, in effect, ranking the two applicants. The first pair of letters (study condition) had mismatched final adjectives (*i.e*., the letter with the stronger performance narrative was coupled with the weaker final adjective and vice versa). For example, the stronger narrative included a weaker final adjective of ‘excellent’ which was comparatively described as placing in the second quintile. The weaker narrative had a final adjective of ‘outstanding’ placing the applicant in the first quintile. Subjects were then to review and rank the second pair of letters (control condition), which had the sentence with the final adjective and the comparative description removed.

**Figure 1.**
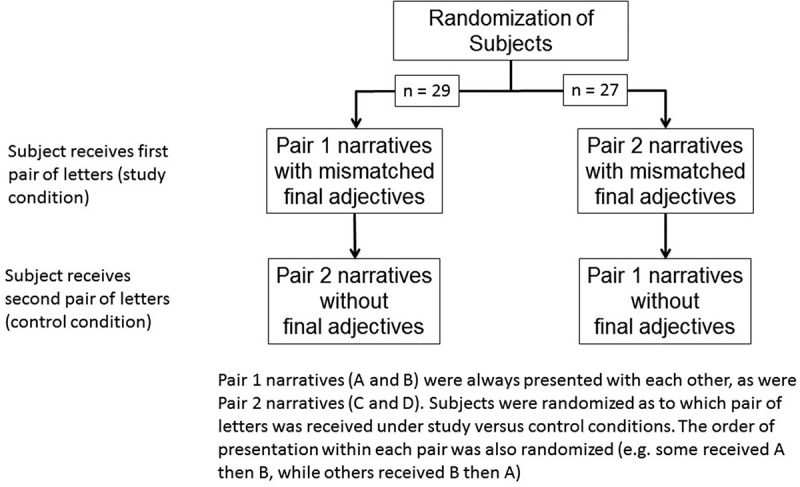
Legend for figure for impact of the final adjective in the Medical Student Performance Evaluation on determination of applicant desirability. Narratives A and B were always paired with each other, as were narratives C and D. Subjects were randomized as to which pair of letters was received under study versus control conditions. The order of presentation within each pair was also randomized (*e.g*., some received A then B, while others received B then A)

To eliminate bias in case the difference between the narratives in one pair was more obvious than in the other pair, subjects were randomized as to whether they received letters A and B with mismatched final adjectives and letters C and D as controls (no final adjective) or C and D with mismatched final adjectives and A and B as controls (Figure 1). Additionally, as there could be a tendency to favor the first or last letter read, the order of presentation of letters within each pair (*i.e*., stronger narrative/weaker adjective or weaker narrative/stronger adjective) was also randomized. After evaluating the first pair of letters, the subjects were given the second pair. Thus, every faculty member reviewed both pairs of letters (*i.e*., all four letters). Half of the subjects received the MSPEs A and B with mismatched final adjectives followed by the MSPEs C and D with the final adjectives removed. The other half received MSPEs C and D with mismatched final adjectives followed by the MSPEs A and B with the final adjectives removed. The pair of letters with mismatched final adjectives was always presented first so that when subjects reviewed the second set (control) and realized that there were no final adjectives, they had no choice but to evaluate based on the narratives. Had the subject been given the pair with no final adjective first, when receiving the second pair with final adjectives he or she might realize the nature of the study and be influenced to pay more attention to the narratives.

Data analysis was conducted with SYSTAT 13, version 1 (SYSTAT Richmond, CA). When comparing two or more proportions, the Pearson chi-square test was used. If the sample size in any group was five or less, a Fisher exact test was performed. The alpha level for all analyses was set at 0.05, and a two-tailed test of significance was used for all calculations. The study was conducted with approval of the BCM Institutional Review Board for Human Research Protocols.

## Results

A total of 56 of 60 invited faculty members agreed to participate Thirty-seven participants (66%) were from the Department of Pediatrics and 19 (34%) from the Department of Medicine; 28 (50%) were male and 28 (50%) female; 11 (20%) were full professors. The majority (61%) had more than 3 years of experience on an intern selection committee.

The proportion of rankings congruent with the strength of the narratives under study and control conditions were compared. When ranking the applicants whose MSPEs had the mismatched final adjectives, subjects preferred the candidate with the stronger adjective (even though the narrative was weaker) the majority of the time (57.1%; 32). When ranking applicants whose letters had narratives only, with no final adjectives, most (82.1%; 46) subjects ranked the applicants congruent with the strength of the narratives. When comparing whether or not the faculty member correctly assessed the ranking of each pair of MSPEs, subjects were significantly more likely to rank the applicants incongruently when reviewing letters in which the strength of the final adjectives conflicted with the strength of the narrative (*χ*^2^ = 18.4 with 1 degree of freedom, *p* < 0.0001 (*p* = 0.001)). There were no significant differences in accuracy of rank order by subject specialty (pediatrics or medicine), gender, academic rank, or years of experience as an interviewer. None of the subjects reported feeling rushed.

## Discussion

Under the conditions of our study, for a majority (57.1%) of subjects the MSPE final adjective had a greater impact than the narrative description of clinical performance on the ranking of applicant desirability. This was despite the fact that the applicants were from different schools, making comparison based on their class quintile problematic.

A study by Loftus *et al*. compared the performance of first-year residents with their reported performance as students on clinical rotations and with their medical school class ranks [5]. The authors concluded that a student’s clinical performance had a stronger relationship to performance as a resident than did the student’s class rank. Weissman has proposed that clerkship evaluation reports (narrative descriptions) were the critical elements in the MSPE [7]. If Loftus [5] and Weissman [7] are correct, and the student’s clinical clerkship performance record is a better predictor of residency performance than the student’s class rank, it is problematic that a majority (57%) of our subjects put more weight on the final adjective than on the narrative description of the clerkship performance. We propose that the two most likely reasons for this are: (1) the relative ease of ranking by final adjective (in this case, quintile); (2) a belief by reviewers that clerkship performance descriptions are generally difficult to interpret. If the prime reason is ease of ranking by final adjective, we hypothesize the inclusion of a final adjective describing the student’s performance relative to his peers will diminish the attention paid by reviewers to the narrative descriptions of performance in clinical clerkships and thereby diminish the usefulness of the narratives. If the prime reason is a belief that clerkship evaluations are difficult to interpret, this would support arguments for a uniform and transparent system of evaluation of student performance on clinical clerkships [7,8]. We agree with the AAMC recommendation that narratives be unedited for content.

In 2007, Lurie and colleagues, surveyed residency program directors in regards to the performance of the graduates of a single US medical school, comparing the director’s perception of the resident’s performance with the student’s rankings in his or her MSPE. The authors concluded, ‘Dean’s letter rankings are a significant predictor of later performance in internship.' [9]. In 2008, a review of MSPEs from 106 US medical schools found that while 80% followed the recommended format, only 17% provided comparative data such as quartiles or a specified hierarchy of categories such as outstanding, excellent, very good, and good [10]. In an additional 30%, comparative data were provided, but the information required to interpret the category was in the appendices rather than in the letters themselves. However, in a review of the literature in 2011, Harfmann and Zirwas concluded that the relationship between the MSPE and performance during residency was unpredictable [11].

The insistence of the 2016 AAMC recommendations that any final adjective be accompanied by a specific explanation of how it was determined is especially important in view of the high reliance on the final adjective found in our study.

In light of our findings of undue reliance on the final adjective, we agree with the AAMC recommendations that: (1) a final adjective or overall rating be provided only if accompanied by a school-wide comparison with the school’s students; (2) information used to rank students be stated clearly; (3) comparative data be provided in the body of the MSPE eliminating Appendices A–D; (4) the MSPE provides information on how final grades and comparative data are derived.

## Limitations

There are several limitations to our study. Our subjects had to read and evaluate only four letters and compare (rank) each letter with the other in the pair. In reality, faculty members usually have to evaluate a larger number of letters and rank each against all the others. In the study, subjects had set aside a more than adequate amount of time to read and rank the four letters; none of the subjects reported feeling rushed. In reality, reviewers may have significant time constraints. We hypothesize that if pressed for time, readers would be even more likely to rely on the final adjective, and so our study may have underestimated the percentage of readers relying on the final adjective. Another limitation is representation from only two disciplines at one medical school. We did not provide grades, and it is possible that grades might influence reviewers more than either the narrative descriptions of clerkship performance or class rank. Finally, although we attempted to address and adjust for all biases, we recognize that there is no way to anticipate all biases that the reviewers may have had.

## Conclusions

The MSPE serves as an evaluation of student performance, and the AAMC has created guidelines to enhance the value of this letter. Under study conditions, where reviewers of MSPEs were presented with mock letters from different schools with mismatched final adjective (indicating class standing by quintile), the majority of reviewers were misled by the mismatched final adjective. Our finding that the final adjective had greater impact than the narrative description of clinical performance on the determination of applicant desirability suggests the need for further standardization or other reforms in the narrative reporting of clerkship evaluations.

## Prior publication

This manuscript has not been published elsewhere, nor is it currently submitted for consideration of publication elsewhere.

## Prior poster presentation

1. Pediatric Academic Societies Annual Meeting, Vancouver, Canada. May 2014.
2. Association of Pediatric Program Directors Spring Meeting, Chicago, IL. April 2014.

## Appendix. Examples of Medical Student Performance Evaluations with mismatched final adjectives

Example A. Stronger Narrative with Weaker (mismatched) Final Adjective

**Medical Student Performance Evaluation**

**IDENTIFYING INFORMATION**

Mark Roberts is a fourth-year medical student at the Medical College of**∎∎∎∎∎∎∎**.

**UNIQUE CHARACTERISTICS**

Mr Roberts is a graduate of the State University of **∎∎∎∎∎∎∎**, where he earned a Bachelor of Science Degree in Biological Sciences. During his time here at the Medical College of **∎∎∎∎∎∎∎**, he has been involved in several research projects as well as a number of extra-curricular service activities, both within his class and outside the school in the local community. Mr Roberts also has participated in two international medical missions, one in Mexico and one in Peru.

**ACADEMIC HISTORY**

Date of expected graduation from medical school: May 2013

Date of initial matriculation in medical school: May 2009

Was this student required to repeat or otherwise remediate any coursework during his/her medical education? No

Was this student the recipient of any adverse action(s) by the medical school or its parent institution? No

**ACADEMIC PROGRESS**

**Preclinical/basic science curriculum**

Mr Roberts completed the first 2 years of the medical school curriculum without difficulty or interruption. He was successful on his first attempt at the USMLE Step 1, with a score of 245 and proceeded to the third year clerkships.

**Core clinical clerkships and required sub-internship rotations**

**Comments regarding this student’s performance in his clinical clerkships are presented below in their entirety and unedited, in chronologic order.**

Medicine

Mr Roberts is a very bright, highly motivated, dedicated, and responsible student. He was able to perform an accurate and thorough history and physical examination and present the data in a clear, concise, and well-organized manner, even on complex and difficult patients. He was at ease with patients, quickly establishing effective rapport. He displayed a fund of knowledge far above that expected for his level of experience. He adjusted quickly to the ways in which the ward functioned and became a valued part of the team – helpful and reliable. Mark researched patient care issues on his own and brought pertinent information to rounds. He was always respectful to patients and to the staff. He actively sought feedback about his performance and used that feedback to further hone his already impressive clinical skills.

Specific comments about Mr Roberts included the following: ‘Very hard worker. Arrived early and stayed late.’ ‘Intelligent, with a strong fund of knowledge. Remarkably efficient and well organized for a core student.’ ‘A hard worker who performed exceedingly well without any problems.’ ‘Great attention to details.’ ‘Fund of knowledge way above average, and he applies this knowledge to the clinical situation without difficulty.’ ‘Handled a full student load and then some. I was surprised to learn this was his first core rotation.’

Surgery

This student did exceedingly well on this rotation. He worked hard, was capable and at ease in the clinical setting and learned quickly. He followed his patients carefully and was always up to date on their conditions. He was energetic and proficient in everything he did. He was always prepared for rounds and presented exceptionally well. Mark was very helpful in the OR, where he was able to provide the staff with current and detailed information about his patients. The residents and the staff felt that Mark will make an excellent physician and commented that they would love to see him become a surgeon.

Family Medicine

Mark is great with patients and performed exceptionally well throughout the rotation. He was always prompt, polite, and professional. He quickly grasped the concept of family care and seemed to appreciate our holistic approach to the patients. His notes were organized and complete, and his presentations when checking out to the attending were equally well organized and complete. His fund of knowledge seemed much better than most students at his level.

Pediatrics

Mark’s overall performance was excellent. He was a talented, hardworking, and enthusiastic self-starter who demonstrated a strong fund of knowledge and equally strong intellectual curiosity. He carried out all assignments and duties in a timely and professional manner and was very involved in the care of his patients. His case presentations were complete, accurate, and well organized, reflecting a remarkably mature and sophisticated understanding of his patients. His patient assessments were always thorough, carefully thought out, and right on target. The residents commented that Mark was very organized and very much a team player.

Psychiatry

Mark is bright, motivated, and energetic. He was at ease in the psychiatric milieu and quickly learned how to conduct a diagnostic interview. He has strong communication skills and interacted well with his patients and with the team. His instructors felt that Mark was very helpful in delivering good patient care during the rotation. Mark obviously did a great deal of reading about his patients and their diagnoses. He is a gifted student and it was truly a pleasure to work with him.

Obstetrics/gynecology

This student’s clinical performance was very strong and entirely without problems. He was pleasant, punctual, and hardworking and always had a positive attitude. He followed his patients carefully and functioned well as part of the team. He not only read about his patients but also consistently searched the literature for information that could be of help in their care and was quite astute in selecting and analyzing data that was evidence-based and pertinent to his patients. Mark was often the first one to notice a new problem or a change in status of one of his patients. His fund of knowledge was very impressive. Mark had an excellent bedside manner and got along well with the physicians and nurses as well as with the patients.

Neurology

Mr Roberts’ performance was exemplary. He was able to do a careful and detailed neurological examination as well as (if not better than) the medicine residents rotating through the neurology service, and he almost always was correct in interpreting the results of the exam. Mark has a wonderful bedside manner – friendly, comforting and professional. His presentation on CNS hemorrhage was very detailed and interesting. Mark is one of the best students to come through our service and we would be delighted to see him go into neurology.

Sub-internship

Ours is a very busy general medicine in-patient unit with many high acuity and complex patients. Mark functioned remarkably well as a sub-intern, seeming to thrive on the fast pace and challenge of caring for many very sick patients. He followed his patients closely, took meticulous care of them, was always current on all details of their problems, and kept the residents informed of their progress. He carried a full load of patients (comparable to that of the interns) and gladly took on some of the most difficult patients. He displayed a clear understanding of what was going on with his patients and was able to organize complex data into a cohesive assessment and treatment plan with a strong sense of priorities. The residents felt that Mark had outstanding clinical skills and was completely reliable and responsible. The attendings who worked with him commented that he was always up to date with his patients and followed through with suggestions about their care quickly and reliably.

In summary, Mr Roberts came to this school with a strong record of performance and service. He has performed exceedingly well both academically and clinically, while giving generously of his time to the school and to the community. **I am pleased to recommend him as an *excellent* candidate for residency, confident that he will be an asset to any program to which he matches.***

***Outstanding: first quintile***

***Excellent*: second quintile**

***Very good to excellent*: third quintile**

***Very good*: fourth quintile**

***Good*: fifth quintile**

*Bolded wording following the asterisk was removed in the second pair of letters (control condition) presented to the subject.

Example B. Weaker Narrative with Stronger (mismatched) Final Adjective

**Medical Student Performance Evaluation**

**IDENTIFYING INFORMATION**

John Applegate is a fourth-year medical student at the University of **∎∎∎∎∎∎∎** College of Medicine.

**UNIQUE CHARACTERISTICS**

Mr Applegate is a graduate of the State University of **∎∎∎∎∎∎∎**, where he earned a Bachelor of Science Degree, with distinction. While here at the University of **∎∎∎∎∎∎∎**College of Medicine, he has actively participated in numerous extra-curriculum service projects, both within and outside of the school community. John has also been involved in two research studies at the school as well as in several international medical activities abroad.

**ACADEMIC HISTORY**

Date of expected graduation from medical school: May 2013

Date of initial matriculation in medical school: Ma, 2009

Was this student required to repeat or otherwise remediate any coursework during his/her medical education? No

Was this student the recipient of any adverse action(s) by the medical school or its parent institution? No

**ACADEMIC PROGRESS**

**Preclinical/basic science curriculum**

Mr Applegate did very well in the basic science portion of the medical curriculum. He continued his academic success in the second year of medical school. John sat for and passed Step 1 of the USMLE on his first attempt with a score of 246.

**Core clinical clerkships and required sub-internship rotations**

**Comments regarding this student’s performance in his clinical clerkships are presented below in their entirety and unedited, in chronologic order.**

Pediatrics

John’s overall performance was exceedingly strong. He had a well above average fund of knowledge and performed consistently well and without any problems. He was able to perform an age-appropriate medical history and physical examination and formulate an appropriate assessment, differential diagnoses and plan. He related very well to the patients and their family members. His case presentations on rounds were well organized, properly focused, accurate, and complete without being too wordy. His clinical reasoning was clear and logical, with a good sense of priorities. The residents felt that John followed his patients closely and contributed to their care.

Obstetrics/gynecology

A consistently excellent student, who took superb care of his patients, helped the residents, wrote complete and accurate notes and presented well on rounds. He was hardworking and dedicated – came in early and stayed late. John was very interested in patient care. He was extremely enthusiastic about learning, read extensively about his patients, and asked good questions. He searched the literature for information pertinent to the care of his patients and presented his findings to the team. John followed up on all assignments, both clinical and academic, quickly, efficiently, and reliably. He was well mannered and friendly to patients, staff, and colleagues. It was a pleasure to have him on our service.

Medicine

Mr Applegate was consistently thorough and well organized. He was able to gather relevant information, analyze it, and formulate an appropriate treatment plan. He showed a better than average fund of basic medical knowledge and was able to apply that information appropriately to an understanding of his patients. He was clearly very interested in learning and responded to feedback quickly and appropriately. John cared for several complicated and seriously ill patients without difficulty, carefully following through on all aspects of their care. He functioned as a highly effective member of the team, earning the respect of the residents and nurses, as well as his patients. He showed unusually strong clinical judgment, quickly recognizing significant changes in the condition of his patients.

Specific comments about Mr Applegate included the following: ‘Did an excellent job. He was reliable and professional and interacted appropriately with the patients, his peers and the ward team. His case write ups were well organized and complete.’ ‘John is a good worker with a great bedside manner and a strong work ethic.’ ‘This student is a quick learner and it was a real pleasure to work with him. Extremely thorough with history taking and did an outstanding job with a very difficult patient. Good fund of knowledge, clearly above average.’

Family Medicine

John is a very enthusiastic and energetic student who participated actively in the clerkship. He saw as many patients as possible, was willing to stay late to see drop-ins, and took advantage of every learning opportunity. He showed great initiative, frequently tracking down lab reports and filling out paperwork without being asked. He has a great work ethic and his maturity and professionalism far exceed that of his peers. Additionally, John has an excellent fund of medical knowledge, which he applies easily to the care of his patients.

Neurology

Mr Applegate worked hard and quickly learned to perform a proficient neurologic history and physical exam. He seemed especially adept at recognizing the difference between pathology and normal variation. His patient work-ups were accurate and thorough, and very well presented, in an orderly and logical manner. John was extremely helpful in caring for his patients, and the residents and nurses considered him a valuable team member. His topic presentation on ALS was appropriately paced and properly focused. We all enjoyed working with him and would be delighted to see him in neurology.

Psychiatry

John was very enthusiastic about the rotation and quickly became involved in the care of his patients. He had a relaxed and professional manner in talking with patients and displayed very effective interviewing skills. He was very conscientious about patient care, spending a great deal of time on the phone and on the computer, making arrangements for their care and facilitating their transition from inpatient to outpatient care. Several instructors and nurses commented that John was an asset to the team.

Surgery

This student was eager, involved, and caring. He was clearly very interested in learning and in participating in patient care. He was well organized and very efficient. On rounds, he knew his patients well, was up to date on their progress, and his presentations were always complete and properly focused. He was prompt, professional, and helpful in the operating room. John’s fund of knowledge seemed considerably above average and he quickly developed an understanding of general surgical principles. He got along very well with the staff and the residents noted that he established good rapport with the patients.

Sub-internship

John performed admirably as a sub-intern. He followed his patients very closely and carefully and was always up to date about their problems and progress. It was clear from his discussions on rounds that he was reading about his patients and looking for information that would help with their care. The residents noted that he did as much as he could on his own but always stayed within the bounds of a sub-intern, asking for help when needed and proceeding on his own then informing the residents when appropriate. He was very conscientious, and the residents considered him an integral part of the team. He was able to manage a full patient load without getting frazzled or falling behind. His clinical skills progressed quickly and he rapidly developed a keen sense of priorities when formulating patient management plans. One of his attendings commented that John had a mature understanding of his patients, was able to see both the forest and the trees, and contributed to their care in very meaningful ways.

In conclusion, Mr Applegate came to this school with an accomplished record of performance and service. He has performed remarkably well both academically and clinically, while giving freely of his time to the school and to the community. **I am pleased to recommend him as an *outstanding* applicant for residency training, confident that he will be an asset to any program to which he matches**. *

***Outstanding: first quintile***

***Excellent*: second quintile**

***Very good to excellent*: third quintile**

***Very good*: fourth quintile**

***Good*: fifth quintile**

*Bolded wording following the asterisk was removed in the second pair of letters (control condition) presented to the subject.

Example C. Stronger Narrative with Weaker (mismatched) Final Adjective

**Medical Student Performance Evaluation**

**IDENTIFYING INFORMATION**

Robert Walters is a senior medical student at the School of Medicine.

**UNIQUE CHARACTERISTICS**

Mr Walters graduated with honors from the University of **∎∎∎∎∎∎∎**, receiving a Bachelor of Science Degree in the Biological Sciences. Here at the School of Medicine, Bob has been involved in numerous extra-curricular activities. He was instrumental in organizing a project for the medically underserved locally and participated in several medical missions abroad. He also has had an active role in two research projects.

**ACADEMIC HISTORY**

Date of expected graduation from medical school: May 2013

Date of initial matriculation in medical school: May 2009

Was this student required to repeat or otherwise remediate any coursework during his/her medical education? No

Was this student the recipient of any adverse action(s) by the medical school or its parent institution? No

**ACADEMIC PROGRESS**

**Preclinical/basic science curriculum**

Mr Walters did extremely well in the basic science portion of the medical curriculum. He continued his academic success in the second year of medical school. He sat for, and easily passed, Step 1 of the USMLE with a score of 255.

**Core clinical clerkships and required sub-internship rotations**

**Comments regarding this student’s performance in his clinical clerkships are given/presented below in their entirety and unedited, in chronologic order.**

Obstetrics/gynecology

Mr Walters’ clinical performance was superb. His histories and physical examinations were well organized and detailed. On rounds, he asked intelligent and insightful questions. He read extensively about his patients, was able to identify what was important and report back to the team on rounds. In addition to outstanding clinical skills, Bob is a strong patient advocate, who puts the needs of his patients and the team before his own. He is the consummate student and his talent and devotion to medicine will make him a great physician. The team will miss him.

Pediatrics

Bob’s performance was outstanding. He had a wonderful way with children and their parents. He has a calm, gentle, and reassuring manner, which quickly puts the child and parents at ease. He is an effective communicator and a fantastic listener, able to obtain, organize and interpret historical data from children and parents. His physical examinations were performed in a friendly yet professional manner and his notes were extremely complete and accurate. He demonstrated a strong fund of knowledge and unusually mature problem-solving skills. The residents felt that Bob was performing at the intern level.

Psychiatry

His instructors noted that Mr Walters was clearly above average in interviewing skills. He was a relaxed and empathic listener, able to obtain a complete and relevant history. He also was able to accurately synthesize the patient’s story. His case reports were excellent, with appropriate differential diagnoses and proposed treatment plans. He participated actively in case conferences, asking excellent questions and bringing up important points in the patients’ history, including cultural factors that might be playing a role in their behavior. Overall: outstanding.

Surgery

Bob was conscientious and always willing to help. His progress notes were clear and concise and reflected excellent knowledge and understanding of his patients. His calm, thoughtful demeanor was very helpful, both on rounds and in the operating room. It was clear that he read extensively about his patients. Bob was always prepared and organized and presented very well at rounds. He works quickly, yet never seems rushed and gets everything done.

Family Medicine

Bob was an extremely competent student who did exceedingly well in the chaotic setting of an office-based group practice. He was poised and comfortable with a variety of patients, from pediatric to geriatric. He has a very positive attitude and an aptitude for self-directed learning. He is very bright and has a very impressive fund of knowledge. He was enthusiastic, reliable, and always professional. It was a real pleasure working with him. The staff considered Bob one of the best students in the past several years.

Neurology

Bob’s overall performance was truly excellent. He was eager and hardworking, well organized, and efficient. He quickly learned how to perform a detailed neurologic examination, and his ability to apply his knowledge of neuroanatomy to the clinical problems of his patients was impressive. At the end of the rotation, he gave a well-organized and informative presentation on the epidemiology of amyotrophic lateral sclerosis.

Medicine

Mr Walters is an enthusiastic and hard-working student who performed much better than the typical core student. He has a calm and thoughtful manner, which is appreciated on a busy and hectic unit such as ours. He quickly demonstrated an exceptional ability to interpret data about complex patients and to revise his assessment and plan on the fly. He was a tremendous asset to the team and everyone enjoyed working with him, nurses as well as house staff and attending physicians. He sought and used feedback to perfect his clinical skills.

Specific comments about Mr Walters included the following: ‘Bob was a wonderful asset to the team, always up to date, completely reliable and responsible.’ ‘It was an absolute pleasure having this student on the team. He was bright, personable, hard working and always on top of things. He never left anything undone.’ ‘Bob got his own work done efficiently, and then looked for ways to help others.’ ‘An exceptionally strong performance. He will make a terrific house officer.’

Sub-internship

Bob did an outstanding job as sub-intern, easily handling a large number of very sick and very complex patients. He followed his patients meticulously, with excellent attention to detail, on several occasions ferreting out information or noting findings on physical examination that others had missed. He was able to assess his patients and sort through difficult problems, while always managing to see the big picture as well as the details. He had an astute sense of priorities when dealing with multiple problems. He has an upbeat yet professional manner with patients, and it was clear that the patients (as well as the residents) saw him as an integral and important part of the team. Several families commented on his reassuring manner and his ability to explain things in a way that they could understand. Bob is truly a star who will become a superb physician, the kind you would want to take care of you or your family.

In summary, Mr Walters came to this school with a strong record of performance and service. He has performed exceedingly well both academically and clinically, while giving generously of his time to the school and to the community. **I am pleased to recommend him as an *excellent* candidate for residency, confident that he will be an asset to any program to which he matches**. ***

***Outstanding*: top 20%:**

***Excellent*: second 20%:**

***Very good*: next 50%:**

***Good*: next 10%:**

*Bolded wording following the asterisk was removed in the second pair of letters (control condition) presented to the subject.

Example D. Weaker Narrative with Stronger (mismatched) Final Adjective

**Medical Student Performance Evaluation**

**IDENTIFYING INFORMATION**

Samuel Lester is a fourth-year medical student at the College of Medicine.

**UNIQUE CHARACTERISTICS**

Mr Lester is a graduate of the University of, where he earned a Bachelor of Science Degree in Biological Sciences. During his time here at the College of Medicine he has been very active in a number of extra-curricular service projects, both within his class and outside the school circle in the local community. During his first summer break Mr Lester participated in a laboratory research project that led to a publication with him as third of six authors.

**ACADEMIC HISTORY**

Date of expected graduation from medical school: May 2013

Date of initial matriculation in medical school: May 2009

Was this student required to repeat or otherwise remediate any coursework during his/her medical education? No

Was this student the recipient of any adverse action(s) by the medical school or its parent institution? No

**ACADEMIC PROGRESS**

**Preclinical/basic science curriculum**

Mr Lester excelled in his preclinical basic science courses. He passed all courses without difficulty, excelled in his ability to apply basic science to clinical problems, and scored 252 on the USMLE Step 1.

**Core clinical clerkships and required sub-internship rotations**

**Comments regarding this student’s performance in his clinical clerkships are given/presented below in their entirety and unedited, in chronologic order.**

Medicine

Mr Lester was considerate, compassionate, humble, and genuinely interested in doing his best to look after his patients. He displayed a friendly and reassuring bedside manner and established strong, professional relationships with his patients. He was a very hard worker and an excellent team player, reliably helpful in caring for all the patients on the team, not just his own. He was very enthusiastic about learning as well as about caring for patients, and frequently went to the literature for answers to questions about the patients.

Specific comments about Sam included the following: ‘He came in early and stayed late and was always ready to pitch in.’ ‘This student is clearly ahead of his classmates in both knowledge and skills, and overall, his performance was outstanding.' ‘Very strong performance. He is very hard working and committed to patient care as well as to his own education.’ ‘Great team player. Great attention to detail.’ ‘His notes reflected a clear understanding of his patients and their progress.’

Family Medicine

Sam was an excellent student who easily fit into the family medicine milieu. He worked well with patients of all ages, establishing rapport, obtaining an accurate history, performing an appropriate physical examination, and generating a comprehensive problem list. He demonstrated sound clinical reasoning. He showed considerable initiative but always verified his plans with staff before implementing them. Excellent communication skills and patient-physician interaction. An outstanding performance without any problems.

Psychiatry

Instructors noted that Sam was very interested in learning and worked hard. He displayed far above average skills in working with psychiatric patients and was at ease interviewing, formulating a diagnostic list and suggesting a treatment plan. He was able to present his patients in a clear and well-organized manner and wrote excellent, detailed case reports. On the inpatient service he volunteered to present one of his patients at our weekly case conference, and in clinic he was always willing to see another patient, including those who came late. Overall: outstanding.

Surgery

Sam is a delightful student, eager to learn and to help. In the OR he was very helpful in talking to the patients and helping them relax before their procedures. On the ward he followed his patients closely and was very attentive to their needs. He was especially helpful in caring for some patients with difficult wounds and painful dressing changes. In the clinics he was equally helpful, gathering all relevant data and presenting his patients in a clear, well-organized manner. He is organized, efficient, and thorough.

Obstetrics/gynecology

Mr Lester’s clinical performance was consistently excellent. He was a very hard worker and entirely reliable. He related well to his patients, followed them closely, and made meaningful contributions to their care. The staff noted that he was especially calming and reassuring to women in labor, both during labor and during the delivery. His level of general medical knowledge was excellent and he quickly accrued a very respectable knowledge of obstetrics and gynecology. He worked well as part of the ward teams during both the obstetrical and the gynecologic parts of the rotation.

Pediatrics

Sam is a diligent and hard worker, who is dependable and always well prepared. The residents and attendings were impressed with his level of initiative and motivation as well as with his clinical skills, and they commented on his maturity and professionalism on more than one occasion. He is bright, with an excellent fund of knowledge, and learns quickly. His patient work-ups were thorough and complete, and were always carefully done. It was clear that he does a lot of outside reading. It was a real pleasure working with Sam. The residents noted that he was definitely an asset to the team and that he will make an excellent physician.

Neurology

Sam’s overall performance was quite strong. He not only demonstrated the ability to perform the neurologic examination, but he also demonstrated the ability to correlate the examination findings with the patients’ lesions and/or diagnoses. Great work ethic and exceptionally reliable. He was always prompt and prepared and followed his patients very closely. At the end of the rotation, he presented an outstanding talk on craniopharyngioma.

Sub-internship

Sam performed exceedingly well as a sub-intern. He quickly took ownership of his patients and followed them closely, analyzing their problems, generating appropriate differential diagnoses, and coming up with his own treatment plans, but he always checked with the resident or supervising attending before writing any orders. He was always prepared and organized and presented well on rounds. Sam asked appropriate questions about his patients but also read about his patients extensively and reported back to the team with helpful information and suggestions. He was quite capable of independent thinking but always stayed within the bounds of his role as a sub-intern. He worked well as part of the team, assisting in the care of all patients as needed, as well as helping to cover for interns who were post-call or had continuity clinic, and receiving high praise from both the house staff and the nurses.

In summary, Mr Lester came to this school with a strong record of performance and service. He has performed exceedingly well both academically and clinically, while giving generously of his time to the school and to the community. **I am pleased to recommend him as an *outstanding* candidate for residency, confident that he will be an asset to any program to which he matches**.*

***Outstanding*: top 20%:**

***Excellent*: second 20%:**

***Very good*: next 50%:**

***Good*: next 10%:**

*Bolded wording following the asterisk was removed in the second pair of letters (control condition) presented to the subject.
